# Current Developments in Detection of Identity-by-Descent Methods and Applications

**DOI:** 10.3389/fgene.2021.722602

**Published:** 2021-09-10

**Authors:** Evan L. Sticca, Gillian M. Belbin, Christopher R. Gignoux

**Affiliations:** ^1^Human Medical Genetics and Genomics Program and Colorado Center for Personalized Medicine, University of Colorado Anschutz Medical Campus, Aurora, CO, United States; ^2^Institute for Genomic Health, Department of Medicine, Icahn School of Medicine at Mount Sinai, New York, NY, United States

**Keywords:** genetics, pedigree, relatedness inference, biobank, identity-by-descent

## Abstract

Identity-by-descent (IBD), the detection of shared segments inherited from a common ancestor, is a fundamental concept in genomics with broad applications in the characterization and analysis of genomes. While historically the concept of IBD was extensively utilized through linkage analyses and in studies of founder populations, applications of IBD-based methods subsided during the genome-wide association study era. This was primarily due to the computational expense of IBD detection, which becomes increasingly relevant as the field moves toward the analysis of biobank-scale datasets that encompass individuals from highly diverse backgrounds. To address these computational barriers, the past several years have seen new methodological advances enabling IBD detection for datasets in the hundreds of thousands to millions of individuals, enabling novel analyses at an unprecedented scale. Here, we describe the latest innovations in IBD detection and describe opportunities for the application of IBD-based methods across a broad range of questions in the field of genomics.

## Introduction

The rapid growth and increasing availability of biobank-scale datasets has led to their increased utilization in human genetics studies, however, the demographic and evolutionary forces that underly genomic patterns within these data are often overlooked. Biases in sample recruitment has led to underrepresentation of non-European ancestry participants, limiting the scope and broad applicability of medical genomics and precision medicine. Additionally, standard genetic analytical frameworks often overlook the fine-scale population structure relevant to the segregation of rare variants, despite their role in common, complex diseases becoming increasingly apparent (Hernandez et al., 2019; Taliun et al., 2021). For these reasons, there is an increasing need for novel methods that can account for demographic substructure driving patterns of variation across the site frequency spectrum in large, diverse cohorts (Gravel et al., 2011). The principle of identity-by-descent (IBD) offers a framework through which we can interpret and leverage the demographic histories of large-scale human genomic data, and improve statistical power to detect causal variants.

Identity-by-descent is the shared inheritance of an identical portion of the genome between two individuals (Browning, 2008; Gusev et al., 2008; Browning and Browning, 2010, Browning and Browning, 2012; Henn et al., 2012; Thompson, 2013). This is distinct from identity-by-state (IBS), in which a portion of two individual’s genomes may appear identical, but not necessarily due to recent shared co-inheritance. Leveraging properties of IBD allows researchers to infer a vast amount of information about a population’s demographic history (Carmi et al., 2013; Palamara and Pe’er, 2013; Nait Saada et al., 2020), allowing for evolutionary and pedigree-derived insights that can aid in the interpretation of genetic variation. Further, identifying these shared segments from a recent common ancestor can enrich for shared patterns of rare variation, due to the relationship between allele age and frequency (Slatkin and Rannala, 2000). In essence, inference of IBD sharing at the population level can allow for the same genetic frameworks behind pedigree studies and linkage analyses to be applied to large population-level genotyped or sequenced data sets. In this review, we explore the population genomic principles governing patterns of IBD sharing, past and recent methods for detecting IBD in population scale data, and downstream applications in contemporary human genomics.

## Governing Evolutionary Population Genetics Principles

Methods of IBD detection, or the identification of haplotypes likely to arise from a recent common ancestor are well established in theory but are rarely applied to modern, biobank-scale datasets. These modern algorithms have been shown to have high accuracy and quick computational run times (Ramstetter et al., 2017). The underlying principle is that long haplotypes shared between individuals are statistically more likely to arise from relatedness due to deep, shared population history as opposed to random recombination or mutation (Browning, 2008; Browning and Browning, 2015). The more closely related individuals are, the higher the percentage of their genome will be shared IBD, since they share a common ancestor more recently in their genealogical history than two randomly sampled individuals. As populations both diverge and intermix over time, lengths of IBD segments will degrade due to recombination (Carmi et al., 2013; Palamara and Pe’er, 2013), therefore longer haplotypic segments tend to represent more recent relatedness due to there being a lower probability of recombination inducing a decay in their length over shorter spans of genealogical time (Henn et al., 2012). For a given set of observed genetic data and associated recombination rate estimates, the unknown population history can be modeled by the population genetics principle of the coalescent. This results in an abundance of information that can be inferred from the properties of the shared IBD segments. The length of a shared IBD segment serves as a proxy for age of the most recent common ancestor at that genomic region, i.e., a longer IBD segment reflects a more recent common ancestor. Therefore, by using IBD to measure local relatedness between individuals along the genome, it is possible to infer aspects of a population’s demographic history. For instance, factors such as the effective population size over antecedent generations, bottlenecks and subsequent founder effects may be estimated given the distribution of observed IBD in a contemporary population (Browning and Browning, 2015). This has implications at the population level, as represented by patterns of IBD-sharing genome-wide, but can also be informative at specific loci along the genome, and can provide demographic and historical context to loci associated with complex traits. IBD can account for demography of a population for a given risk allele, that is, a variant arising through mutation or recombination, spreading and surviving in a population due to demographic events and genetic drift, has information that is encoded in the spanning inherited segment that is informative of evolutionary and complex disease processes (Nelson et al., 2018; Tian et al., 2019). With the concept of IBD explained, we will now offer some of the applications in contemporary human genomics.

A crucial goal in population genetics is the estimation of the mutation rate across the genome. IBD-based methods can augment mutation rate estimation approaches by leveraging IBD segments to condition on recent ancestry as part of the estimation process. Prior techniques involved using trios of parents and offspring to estimate mutation rate. However, this approach is difficult to implement due to the logistical challenges of recruiting trios, and is sensitive to genotyping errors or somatic mutations being incorrectly classified as *de novo* mutations (Shah et al., 2018; Tian et al., 2019). In identifying IBD segments, researchers can quantify the *de novo* mutation rate on each segment related to the degree of kinship between the samples to reduce the false positive rate, particularly when compared to small pedigree-based studies. Furthermore, IBD methods allow for the expansion beyond pedigree studies to large-scale population-based datasets by leveraging the inherent background IBD present in human populations, with recent investigations further narrowing the confidence in our estimation of mutation rates to between 1.02 × 10^–8^ and 1.56 × 10^–8^ (Campbell et al., 2012; Palamara et al., 2015). Other studies have shown that inferring short IBD segments into longer IBD segments can help to adjust estimations of the *de novo* mutation rate (Chiang et al., 2016). By leveraging IBD, the fundamental question of what mutation rates are across the genome can be more confidently assessed by creating more complete models of mutation, recombination and kinship.

Alongside interrogating the mutation rate of the genome, there has been significant interest in determining the variation in the recombination landscape among global human populations. In addition to having different population level prevalences, the same complex disease loci may exhibit local differences in linkage disequilibrium that directly impact fine-mapping and other common genetic analyses (Wojcik et al., 2019). This means that population-specific recombination maps will be important for fine-mapping both common and rare variants in complex diseases in diverse populations. One recent study showed that building a recombination map from IBD segments yields better estimation of recombinational endpoints and time-to-most-recent-common-ancestor when compared to LD- or admixture-based approaches (Zhou et al., 2020a). Here, IBD methods, particularly those that can work accurately and at scale, can help to create population specific recombination maps that will in turn allow for more accurate simulations of each specific population’s demographic history, leading to other downstream applications such as improved imputation.

Identity-by-descent detection also plays into the recent advances in population structure estimation, particularly at fine scale. Inherent to the idea of a population is the idea of shared ancestry and with this shared ancestry comes a higher probability of relatedness, and a larger portion of the genome shared IBD between any sampled individuals within the same population, when compared to two individuals sampled from between populations. We consider, as an example, the question of improving admixture inference accuracy. By identifying IBD segments among individuals in a population, admixture measurements can be considered with higher accuracy than just comparing genotypes, which may be additionally influenced by errors or somatic mutations. In addition, as studies grow larger, the search space for identifying shared cryptic ancestry as captured by IBD tends to scale quadratically (i.e., with the total pairs of individuals). Thus, a high degree of cryptic relatedness can be present in large-scale genetics studies when a prior, smaller study in the same population may have shown little to no cryptic relatedness. To account for this component of population structure, IBD methods allow researchers to reduce confounding in their study design and better reflect the populations’ allele frequencies by matching cases and controls on the basis of genetic ancestry (Palin et al., 2011; Nelson et al., 2018; Sohail et al., 2019).

Concurrent with GWAS, mapping of genetic variants to IBD segments and/or clusters is an alternative method that can help to detect significant associations with a trait of interest. This is similar to how the technique of linkage mapping narrows the genetic signal to a linkage peak (Gusev et al., 2011; Browning and Thompson, 2012). Rare, causal variants preserved in the population while being affected by population demography, drift, selection and substructure have been shown to fall within segments of the genome that are IBD between pairs of individuals in study populations. Analysis of founder populations offer examples of how rare variants can be identified using IBD methods: one example showed how broadly rare European variants contribute disproportionately to disease risk in Quebec (Nelson et al., 2018). Similarly, the elevated IBD patterns present in island populations have empowered novel discoveries, such as the link between height-associated loci and a collagen disorder found in Puerto Ricans (Belbin et al., 2017). With increasing recognition of the role of rare variants in complex disease, and the highly structured manner in which they segregate, methods that leverage IBD for rare variant detection have the potential to be increasingly useful for rare variant discovery.

Finally, imputation can be dramatically improved when leveraging the population specific information inherent to IBD. With growing reference panels from global populations, imputation is resulting in more accurate haplotype matching (Kowalski et al., 2019). IBD can further improve this by noting how to match sample haplotypes to appropriate ancestral references for imputation in a concept called a Study-Specific Reference Panel (SSRP; Gusev et al., 2012; Uricchio et al., 2012; Abney and ElSherbiny, 2019). In practice, modern imputation methods hosted in current servers attempt to approximate this process, but do not recapitulate the augmentation of standard reference panels with appropriate SSRPs (Das et al., 2016). Even without a well annotated pedigree, modern IBD techniques show that imputation quality can be drastically improved when leveraging SSRPs above typical LD based imputation methods (Abney and ElSherbiny, 2019). Not only is IBD useful alone, but it also augments more standard imputation methods by improving imputation probabilities at difficult-to-impute SNPs. By creating custom SSRPs, recruitment efforts to improve representation of understudied populations in human genetics (Bustamante et al., 2011; Popejoy and Fullerton, 2016) can be efficiently leveraged for imputing rare variants, particularly those with greater population-specificity (Gravel et al., 2011).

With the utility of IBD detection outlined, we will next describe the theoretical, statistical and computational means through which IBD detection algorithms are implemented.

## Overview of Methods

Both novel computational paradigms and improvements in computational architecture have led to scalable and accurate methods for IBD detection (Table 1). Originally, whether through strict string pattern matching or fuzzier matching, methods were not equipped to deal with the inherent quadratic scaling of IBD, limiting the size of initial investigations. The era of high-throughput IBD detection began with GERMLINE (Gusev et al., 2008) to detect variation in IBD patterns efficiently and explore how they are influenced by population processes. GERMLINE creates a hash table between short, exact matches of haplotypes and extending into longer, fuzzy (i.e., allowing for small SNP mismatches or genotype errors) IBD segments. This “seed and extend” paradigm, leveraging the inherent efficiency of short hashing functions for speedup beyond standard pairwise comparisons has been adopted by subsequent detection algorithms (Shemirani et al., 2019; Nait Saada et al., 2020), and improved efficiency over hidden Markov model (HMM)-based algorithms or simpler string matching approaches. The computational efficiency garnered by GERMLINE allows computational time to scale approximately linearly with the number of samples and genotyped variants. While GERMLINE demonstrated accuracy and efficiency in identifying known IBD from simulated datasets and early GWAS studies, it does not easily scale to sample sizes in the hundreds of thousands of individuals, as seen in many contemporary genetic cohorts [although it can provide meaningful insights into biobank-scale data with extensive parallelization (Sapin and Keller, 2021)]. Thus, the primary value in detailing GERMLINE is to describe how it influenced the current IBD calling algorithms outlined below. While GERMLINE works in both diploid and haploid modes, much recent work has been focused on recent haploid methods given the ubiquity of phasing in modern genomic analyses, although we discuss recent efforts in diploid IBD detection as well.

**TABLE 1:** Overview of IBD detection tools.

**Tool Name**	**Underlying algorithm**	**Diploid/**	**Citation**	**OS compatibility**	**Link**
		**Haploid**			
GERMLINE	Hash and extension	Diploid/Haploid	Gusev et al., 2008	UNIX, compile with make	http://gusevlab.org/projects/germline
RaPID	PBWT on phased haplotypes	Haploid	Naseri et al., 2019	UNIX, pre-compiled	https://github.com/ZhiGroup/RaPID
ILASH	Locality sensitive hashing, extension	Haploid	Shemirani et al., 2019	UNIX, CMAKE v3.5 or higher required	https://github.com/roohy/iLASH
Phaseibd	Templated positional Burrows–Wheeler transform (TPBWT)	Diploid/Haploid	Freyman et al., 2021	UNIX, requires the Python packages: Cython, numpy, and pandas	https://github.com/23andMe/phasedibd
FastSMC	Hash/Extend plus HMM for validation	Haploid	Nait Saada et al., 2020	Ubuntu, macOS, compile with cmake, some python dependencies	https://github.com/PalamaraLab/FastSMC
IBIS	Sliding Window Overlap on unphased genotypes	Diploid	Seidman et al., 2020	UNIX, compile with make	https://github.com/williamslab/ibis
Hap-IBD	Error adjusted PBWT	Haploid	Zhou et al., 2020b	UNIX, runs with java -jar	https://github.com/browning-lab/hap-ibd

*PBWT, Positional Burroughs-Wheeler Transformation; HMM, Hidden Markov Model.*

One of recent innovations in the rapid detection of IBD segments is the ILASH algorithm (Shemirani et al., 2019). ILASH works on the principle of locality sensitive hashing (Leskovec et al., 2020) to efficiently search the genome. It begins with a similar “seed and extend” hash table of two individuals in a data set via small stretches of DNA and extending data if the two stretches meet criteria matching IBD similarity. The locality sensitive hashing implemented in ILASH is scalable to IBD detection in tens to hundreds of thousands of individuals, such as in the PAGE Study and UK BioBank. Furthermore, it utilizes multiple parallelized computing across multiple stages of the algorithm to ensure optimization. While ILASH is optimized for the biobank era of genetics and proves easy to use in standard analysis pipelines, there are other algorithms with alternative mathematical and computational approaches.

Another solution to efficient IBD detection is RaPID (Naseri et al., 2019). Instead of locality sensitive hashing, RaPID works through random projections of the low-resolution genetic data and applying the Positional Burroughs-Wheeler Transformation (PBWT; Durbin, 2014)between phased individual haplotypes until a perfect match is obtained. These matches are also stored in a hash table and extended with further matches as previously detailed, combining those results into an IBD segment. While PBWT is an efficient data transformation for genetic data, a key additional step in RaPID incorporates the approximate matching needed to be added to tolerate small mismatches, while only adding trivially to the computational time. Furthermore, the accuracy of results can be improved by subsequent iterations of PWBT, albeit at the cost of longer analysis time. Developers also benchmarked RaPID on simulated and UK BioBank data, showing performance and accuracy results similar to those of ILASH.

Another method that has been developed on top of existing theory is hap-IBD (Zhou et al., 2020b). Building on extensive previous work in IBD estimation through the Beagle software program, researchers have made significant advances in haploid IBD speed. In their most recent efforts, they developed hap-IBD as an algorithm for implementing PBWT similar to RaPID. It differs from RaPID in that it controls for false positives of genotype error or mutation by allowing for small gaps of non-IBS between IBD segments. This allows the algorithm to account for gene conversion, a common phenomenon that can disrupt otherwise IBD segments. In addition, hap-IBD may run the PBWT in parallel, thus showing the best performance among algorithms benchmarked in UK BioBank data. Similarly, investigators at 23andMe leveraged the same PBWT to develop their new Templated PBWT framework (Freyman et al., 2021) with similar properties and efficient, scalable runtime. TPBWT is notable for attempting to identify and correct phase switch errors, thereby improving IBD tract length estimation and long-range phasing.

Another novel algorithmic extension that builds on IBD detection and that shows high performance in accuracy as well as speed is FastSMC (Nait Saada et al., 2020). FastSMC builds upon the hash table GERMLINE method as a first identification step by also including a validation step that uses a approximate coalescent HMM (Palamara et al., 2018). This second step distinguishes between segments of IBS and IBD by estimating the probability a shared IBS segment is due to recent common ancestry, thus allowing for IBD calls within shorter windows. This coalescence probability is reported as an IBD quality score, providing a further layer of information in addition to the IBD haplotypes themselves. By implementing this validation step, FastSMC shows higher accuracy in IBD identification at limited additional computational performance when compared to other algorithms. FastSMC is just one of many IBD identification tools that extend upon the frameworks originated in GERMLINE to improve performance and accuracy, and because of its two-step design, it could easily be adapted to utilize one of the newer IBD detection methods to further improve efficiency of the initial step.

While many IBD detection methods rely upon accurate phasing of alleles, one approach, IBIS, does not have this caveat. IBIS works through long range allelic sharing, detecting shared homozygous alleles between individuals and uses Boolean logic operators to determine IBD from a given rule set (Seidman et al., 2020). The main benefit of IBIS compared to other methods is the time and computational resources saved from not having to pre-phase the genetic data before IBD detection. The major caveat behind this is that without phase information providing haplotype resolution, excess homozygosity within putative IBD segments can increase the false positive rate, and the shortest segments detectable in diploid IBD are larger than in haploid methods. However, this limitation on segment length (say ∼7 cM for diploid, versus 2–3 cM for haploid) can be acceptable for certain analyses. As previously stated, more recently related individuals share longer IBD segments which may empower risk allele identification or where measuring the length of long IBD segments is of particular importance. Researchers may be especially interested in IBIS as an intermediate analysis strategy, balancing accuracy and speed, for preliminary exploration of a dataset, or for applications that do not require phasing.

A final value to IBD is that in association studies looking for rare, causal variants in complex disease with large biobank sample sized data sets, IBD offers improved statistical power over traditional GWAS methods. This is because, rare variants are much more likely to be found within an IBD cluster (Nait Saada et al., 2020). Coalescence simulation-based work has shown the concordance between IBD and rare exomic variants (Nait Saada et al., 2020). Similarly, in the UK BioBank, researchers found significant associations to blood related traits otherwise not detected in exome-based tests by using IBD methods to predict sharing of ultra-rare, causal variants (MAF < 0.0001; Nait Saada et al., 2020). By identifying regions of IBD where rare, causal variants are likely to occur, the threshold for significance can be appropriately lowered, analogous to how a linkage peak narrows the search for a genetic signal. As a result of looking for associations between IBD segments and complex disease status, we propose the coining of the term “IBDWAS” to make the value of IBD-driven insights more pronounced.

## Conclusion

To summarize, IBD has significant but often-overlooked meaning in human genetics studies in the context of biobank scale data. All genetic variants affecting traits are influenced by the combination of the evolutionary forces of selection and genetic drift. While in the past inferring the demographic history of a study’s population was difficult, the field of genomics has reached datasets so large that ignoring underlying population history can lead to inappropriate conclusions in disease associations and pathogenicity adjudication. As biobank-scale datasets continue to grow, IBD-based analyses offer a paradigm to address unanswered questions within the field of genomics, and with recent advances in IBD-detection methods there are new opportunities to study these patterns of relatedness at scale. It is therefore relevant to incorporate methods of IBD detection into genetic studies to gain insights into the demographic history of variants of interest, to improve statistical power in detecting rare, causal variants, and to improve the accuracy of imputation, among other relevant analyses.

## Author Contributions

ES initially drafted the manuscript with edits and contributions from GB and CG. All authors contributed to the article and approved the submitted version.

## Author Disclaimer

The content is solely the responsibility of the authors and does not necessarily represent the official views of the National Institutes of Health.

## Conflict of Interest

The authors declare that the research was conducted in the absence of any commercial or financial relationships that could be construed as a potential conflict of interest.

## Publisher’s Note

All claims expressed in this article are solely those of the authors and do not necessarily represent those of their affiliated organizations, or those of the publisher, the editors and the reviewers. Any product that may be evaluated in this article, or claim that may be made by its manufacturer, is not guaranteed or endorsed by the publisher.

## References

[B1] Abney M. ElSherbiny A. (2019). Kinpute: using identity by descent to improve genotype imputation. *Bioinformatics* 35 4321–4326. 10.1093/bioinformatics/btz221 30918937 PMC6821425

[B2] Belbin G. M. Odgis J. Sorokin E. P. Yee M. C. Kohli S. Glicksberg B. S. (2017). Genetic identification of a common collagen disease in puerto ricans via identity-by-descent mapping in a health system. *Elife* 6:e25060. 10.7554/eLife.25060.033 PMC5595434 28895531

[B3] Browning S. (2008). Estimation of pairwise identity by descent from dense genetic marker data in a population sample of haplotypes. *Genetics* 178 2123–2132. 10.1534/genetics.107.084624 18430938 PMC2323802

[B4] Browning S. R. Browning B. L. (2010). High-resolution detection of identity by descent in unrelated individuals. *Am. J. Hum. Genet.* 86 526–539. 10.1016/j.ajhg.2010.02.021 20303063 PMC2850444

[B5] Browning S. R. Browning B. L. (2012). Identity by descent between distant relatives: detection and applications. *Annu. Rev. Genet.* 46 617–633. 10.1146/annurev-genet-110711-155534 22994355

[B6] Browning S. R. Browning B. L. (2015). Accurate non-parametric estimation of recent effective population size from segments of identity by descent. *Am. J. Hum. Genet.* 97 404–418. 10.1016/j.ajhg.2015.07.012 26299365 PMC4564943

[B7] Browning S. R. Thompson E. A. (2012). Detecting rare variant associations by identity-by-descent mapping in case-control studies. *Genetics* 190 1521–1531. 10.1534/genetics.111.136937 22267498 PMC3316661

[B8] Bustamante C. D. Burchard E. G. De la Vega F. M. (2011). Genomics for the world. *Nature* 475 163–165. 10.1038/475163a 21753830 PMC3708540

[B9] Campbell C. D. Chong J. X. Malig M. Ko A. Dumont B. L. Han L. (2012). Estimating the human mutation rate using autozygosity in a founder population. *Nat. Genet.* 44 1277–1281. 10.1038/ng.2418 23001126 PMC3483378

[B10] Carmi S. Palamara P. F. Vacic V. Lencz T. Darvasi A. Pe’er I. (2013). The variance of identity-by-descent sharing in the Wright-Fisher model. *Genetics* 193 911–928. 10.1534/genetics.112.147215 23267057 PMC3584006

[B11] Chiang C. W. K. Ralph P. Novembre J. (2016). Conflation of short identity-by-descent segments bias their inferred length distribution. *G3* 6 1287–1296. 10.1534/g3.116.027581 26935417 PMC4856080

[B12] Das S. Forer L. Schonherr S. Sidore C. Locke A. E. Kwong A. (2016). Next-generation genotype imputation service and methods. *Nat. Genet.* 48 1284–1287. 10.1038/ng.3656 27571263 PMC5157836

[B13] Durbin R. (2014). Efficient haplotype matching and storage using the positional Burrows-Wheeler transform (PBWT). *Bioinformatics* 30 1266–1272. 10.1093/bioinformatics/btu014 24413527 PMC3998136

[B14] Freyman W. A. McManus K. F. Shringarpure S. S. Jewett E. M. Bryc K. Me Research T. (2021). Fast and robust identity-by-descent inference with the templated positional burrows-wheeler transform. *Mol. Biol. Evol.* 38 2131–2151. 10.1093/molbev/msaa328 33355662 PMC8097300

[B15] Gravel S. Henn B. M. Gutenkunst R. N. Indap A. R. Marth G. T. Clark A. G. (2011). Demographic history and rare allele sharing among human populations. *Proc. Natl. Acad. Sci. U.S.A.* 108 11983–11988. 10.1073/pnas.1019276108 21730125 PMC3142009

[B16] Gusev A. Kenny E. E. Lowe J. K. Salit J. Saxena R. Kathiresan S. (2011). DASH: a method for identical-by-descent haplotype mapping uncovers association with recent variation. *Am. J. Hum. Genet.* 88 706–717. 10.1016/j.ajhg.2011.04.023 21620352 PMC3113343

[B17] Gusev A. Lowe J. Stoffel M. Daly M. Altshuler D. (2008). Whole population, genomewide mapping of hidden relatedness. *Genome Res.* 19 318–326. 10.1101/gr.081398.108 18971310 PMC2652213

[B18] Gusev A. Shah M. J. Kenny E. E. Ramachandran A. Lowe J. K. Salit J. (2012). Low-pass genome-wide sequencing and variant inference using identity-by-descent in an isolated human population. *Genetics* 190 679–689. 10.1534/genetics.111.134874 22135348 PMC3276614

[B19] Henn B. Hon L. Macpherson J. Eriksson N. (2012). Cryptic distant relatives are common in both isolated and cosmopolitan genetic samples. *PLoS One* 7:e34267. 10.1371/journal.pone.0034267 22509285 PMC3317976

[B20] Hernandez R. D. Uricchio L. H. Hartman K. Ye C. Dahl A. Zaitlen N. (2019). Ultrarare variants drive substantial cis heritability of human gene expression. *Nat. Genet.* 51 1349–1355. 10.1038/s41588-019-0487-7 31477931 PMC6730564

[B21] Kowalski M. H. Qian H. Hou Z. Rosen J. D. Tapia A. L. Shan Y. (2019). Use of >100,000 NHLBI trans-omics for precision medicine (TOPMed) consortium whole genome sequences improves imputation quality and detection of rare variant associations in admixed African and Hispanic/Latino populations. *PLoS Genet.* 15:e1008500. 10.1371/journal.pgen.1008500 31869403 PMC6953885

[B22] Leskovec J. Rajaraman A. Ullman J. D. (2020). *Mining of Massive Datasets.* New York, NY: Cambridge University Press. 10.1017/9781108684163

[B23] Nait Saada J. Kalantzis G. Shyr D. Cooper F. Robinson M. Gusev A. (2020). Identity-by-descent detection across 487,409 British samples reveals fine scale population structure and ultra-rare variant associations. *Nat. Commun.* 11:6130. 10.1038/s41467-020-19588-x 33257650 PMC7704644

[B24] Naseri A. Liu X. Tang K. Zhang S. Zhi D. (2019). RaPID: ultra-fast, powerful, and accurate detection of segments identical by descent (IBD) in biobank-scale cohorts. *Genome Biol.* 20:143. 10.1186/s13059-019-1754-8 31345249 PMC6659282

[B25] Nelson D. Moreau C. de Vriendt M. Zeng Y. Preuss C. Vezina H. (2018). Inferring transmission histories of rare alleles in population-scale genealogies. *Am. J. Hum. Genet.* 103 893–906. 10.1016/j.ajhg.2018.10.017 30526866 PMC6288464

[B26] Palamara P. F. Francioli L. C. Wilton P. R. Genovese G. Gusev A. Finucane H. K. (2015). Leveraging distant relatedness to quantify human mutation and gene-conversion rates. *Am. J. Hum. Genet.* 97 775–789. 10.1016/j.ajhg.2015.10.006 26581902 PMC4678427

[B27] Palamara P. F. Pe’er I. (2013). Inference of historical migration rates via haplotype sharing. *Bioinformatics* 29 i180–i188. 10.1093/bioinformatics/btt239 23812983 PMC3694674

[B28] Palamara P. F. Terhorst J. Song Y. S. Price A. L. (2018). High-throughput inference of pairwise coalescence times identifies signals of selection and enriched disease heritability. *Nat. Genet.* 50 1311–1317. 10.1038/s41588-018-0177-x 30104759 PMC6145075

[B29] Palin K. Campbell H. Wright A. F. Wilson J. F. Durbin R. (2011). Identity-by-descent-based phasing and imputation in founder populations using graphical models. *Genet. Epidemiol.* 35 853–860. 10.1002/gepi.20635 22006673 PMC3368215

[B30] Popejoy A. B. Fullerton S. M. (2016). Genomics is failing on diversity. *Nature* 538 161–164. 10.1038/538161a 27734877 PMC5089703

[B31] Ramstetter M. D. Dyer T. D. Lehman D. M. Curran J. E. Duggirala R. Blangero J. (2017). Benchmarking relatedness inference methods with genome-wide data from thousands of relatives. *Genetics* 207 75–82. 10.1534/genetics.117.1122 28739658 PMC5586387

[B32] Sapin E. Keller M. C. (2021). Novel approach for parallelizing pairwise comparison problems as applied to detecting segments identical by decent in whole-genome data. *Bioinformatics* 37 2121–2125. 10.1093/bioinformatics/btab084 33705528 PMC8352502

[B33] Seidman D. N. Shenoy S. A. Kim M. Babu R. Woods I. G. Dyer T. D. (2020). Rapid, phase-free detection of long identity-by-descent segments enables effective relationship classification. *Am. J. Hum. Genet.* 106 453–466. 10.1016/j.ajhg.2020.02.012 32197076 PMC7118564

[B34] Shah N. Hou Y. C. Yu H. C. Sainger R. Caskey C. T. Venter J. C. (2018). Identification of misclassified clinvar variants via disease population prevalence. *Am. J. Hum. Genet.* 102 609–619. 10.1016/j.ajhg.2018.02.019 29625023 PMC5985337

[B35] Shemirani R. Belbin G. M. Avery C. L. Kenny E. E. Gignoux C. R. Ambite J. L. (2019). Rapid detection of identity-by-descent tracts for mega-scale datasets. *bioRxiv* [Preprint]. 10.1101/749507 PMC8192555 34112768

[B36] Slatkin M. Rannala B. (2000). Estimating allele age. *Annu. Rev. Genomics Hum. Genet.* 1 225–249. 10.1146/annurev.genom.1.1.225 11701630

[B37] Sohail M. Maier R. M. Ganna A. Bloemendal A. Martin A. R. Turchin M. C. (2019). Polygenic adaptation on height is overestimated due to uncorrected stratification in genome-wide association studies. *Elife* 8:e39702. 10.7554/eLife.39702 30895926 PMC6428571

[B38] Taliun D. Harris D. N. Kessler M. D. Carlson J. Szpiech Z. A. Torres R. (2021). Sequencing of 53,831 diverse genomes from the NHLBI TOPMed Program. *Nature* 590 290–299. 10.1038/s41586-021-03205-y 33568819 PMC7875770

[B39] Thompson E. A. (2013). Identity by descent: variation in meiosis, across genomes, and in populations. *Genetics* 194 301–326. 10.1534/genetics.112.148825 23733848 PMC3664843

[B40] Tian X. Browning B. L. Browning S. R. (2019). Estimating the genome-wide mutation rate with three-way identity by descent. *Am. J. Hum. Genet.* 105 883–893. 10.1016/j.ajhg.2019.09.012 31587867 PMC6848988

[B41] Uricchio L. H. Chong J. X. Ross K. D. Ober C. Nicolae D. L. (2012). Accurate imputation of rare and common variants in a founder population from a small number of sequenced individuals. *Genet. Epidemiol.* 36 312–319. 10.1002/gepi.21623 22460724 PMC3794468

[B42] Wojcik G. L. Graff M. Nishimura K. K. Tao R. Haessler J. Gignoux C. R. (2019). Genetic analyses of diverse populations improves discovery for complex traits. *Nature* 570 514–518. 10.1038/s41586-019-1310-4 31217584 PMC6785182

[B43] Zhou Y. Browning B. L. Browning S. R. (2020a). Population-specific recombination maps from segments of identity by descent. *Am. J. Hum. Genet.* 107 137–148. 10.1016/j.ajhg.2020.05.016 32533945 PMC7332656

[B44] Zhou Y. Browning S. R. Browning B. L. (2020b). A fast and simple method for detecting identity-by-descent segments in large-scale data. *Am. J. Hum. Genet.* 106 426–437. 10.1016/j.ajhg.2020.02.010 32169169 PMC7118582

